# Orally Administered 5-aminolevulinic Acid for Isolation and Characterization of Circulating Tumor-Derived Extracellular Vesicles in Glioblastoma Patients

**DOI:** 10.3390/cancers12113297

**Published:** 2020-11-07

**Authors:** Sybren L. N. Maas, Thomas S. van Solinge, Rosalie Schnoor, Anudeep Yekula, Joeky T. Senders, Jeroen de Vrij, Pierre Robe, Bob S. Carter, Leonora Balaj, Ger J. A. Arkesteijn, Esther N. M. Nolte-’t Hoen, Marike L. D. Broekman

**Affiliations:** 1 Department of Neurosurgery, UMC Utrecht Brain Center, Utrecht University, 3584 CX Utrecht, The Netherlands; s.l.n.maas-3@umcutrecht.nl (S.L.N.M.); rosalie.schnoor@arkin.nl (R.S.); J.T.Senders@umcutrecht.nl (J.T.S.); 2 Department of Pathology, University Medical Center Utrecht, Utrecht University, 3584 CX Utrecht, The Netherlands; 3 Departments of Neurology and Radiology, Massachusetts General Hospital, and NeuroDiscovery Center, Harvard Medical School, Boston, MA 02114, USA; tvansolinge@mgh.harvard.edu; 4 Department of Neurosurgery, Leiden University Medical Center, 2333 ZA Leiden, The Netherlands; 5 Department of Neurosurgery, Massachusetts General Hospital, Harvard Medical School, Boston, MA 02114, USA; AYEKULA@mgh.harvard.edu (A.Y.); BCARTER@mgh.harvard.edu (B.S.C.); Balaj.Leonora@mgh.harvard.edu (L.B.); 6 Department of Neurosurgery, Erasmus MC, 3015 GD Rotterdam, The Netherlands; j.devrij@erasmusmc.nl; 7 Department of Neurosurgery, University Medical Centre Utrecht, 3584 CX Utrecht, The Netherlands; p.robe@umcutrecht.nl; 8 Department of Biomolecular Health Sciences, Faculty of Veterinary Medicine, Utrecht University, 3584 CX Utrecht, The Netherlands; g.j.a.arkesteijn@uu.nl (G.J.A.A.); E.N.M.Nolte@uu.nl (E.N.M.N.H.); 9 Department of Neurosurgery, Haaglanden Medical Center, 2512 VA The Hague, The Netherlands

**Keywords:** glioblastoma, 5 aminolevulinic acid, protoporphyrin IX, extracellular vesicles, high-resolution flow cytometry

## Abstract

**Simple Summary:**

In Glioblastoma (GB), a malignant tumor of the central nervous system, diagnosis can currently only be obtained via tissue biopsy. In this study we were able to isolate GB derived extracellular vesicles (EVs) in the blood, after patients received 5-aminolevulinic acid (5-ALA) before surgery. This isolation is based on fluorescence caused by the accumulation of fluorescent protoporphyrin IX (PpIX) in these EVs. We show that these EVs contain various GB-related micro RNAs. While there are many ways in which our technique needs to be improved before being able to be implemented in the clinic, this study shows that detecting and analyzing circulating GB-derived EVs based on PpIX fluorescence is feasible. In the future, our technique could be developed to diagnose and monitor GB via blood samples instead of a brain biopsy.

**Abstract:**

*Background:* In glioblastoma (GB), tissue is required for accurate diagnosis and subtyping. Tissue can be obtained through resection or (stereotactic) biopsy, but these invasive procedures provide risks for patients. Extracellular vesicles (EVs) are small, cell-derived vesicles that contain miRNAs, proteins, and lipids, and possible candidates for liquid biopsies. GB-derived EVs can be found in the blood of patients, but it is difficult to distinguish them from circulating non-tumor EVs. 5-aminolevulinic acid (5-ALA) is orally administered to GB patients to facilitate tumor visualization and maximal resection, as it is metabolized to fluorescent protoporphyrin IX (PpIX) that accumulates in glioma cells. In this study, we assessed whether PpIX accumulates in GB-derived EVs and whether these EVs could be isolated and characterized to enable a liquid biopsy in GB. *Methods:* EVs were isolated from the conditioned media of U87 cells treated with 5-ALA by differential ultracentrifugation. Blood samples were collected and processed from healthy controls and patients undergoing 5-ALA guided surgery for GB. High-resolution flow cytometry (hFC) enabled detection and sorting of PpIX-positive EVs, which were subsequently analyzed by digital droplet PCR (ddPCR). *Results:* PpIX-positive EVs could be detected in conditioned cell culture media as well as in patient samples after administration of 5-ALA. By using hFC, we could sort the PpIX-positive EVs for further analysis with ddPCR, which indicated the presence of EVs and GB-associated miRNAs. *Conclusion:* GB-derived EVs can be isolated from the plasma of GB patients by using 5-ALA induced fluorescence. Although many challenges remain, our findings show new possibilities for the development of blood-based liquid biopsies in GB patients.

## 1. Introduction

In adult patients, glioblastoma (GB) is the most common primary malignancy of the central nervous system, with a dismal prognosis [1,2]. Even with extensive treatment including maximal neurosurgical resection, chemo- and radiotherapy, median survival is approximately 15 months after initial diagnosis, which has scarcely improved since the introduction of Temozolomide over a decade ago [3].

One of the many challenges faced by GB treatment and research, is that access to the tumor is difficult. Definitive diagnosis still requires tissue, which can only be acquired via (stereotactic) biopsy or neurosurgical resection of the tumor. Although surgical resection is essential to extend the progression-free period in glioblastoma patients, both stereotactic biopsies and more extended surgical approaches are associated with significant morbidity and mortality [4,5,6]. For example, complications were reported after stereotactic intracranial biopsies in 12.1% of cases in one study, with a 3.8% overall mortality [4]. This makes repeated biopsies for follow-up or research purposes currently unjustifiable, even more so as the morbidity and mortality of these procedures increases with successive procedures [7]. There are also concerns regarding the effects of a biopsy on tumor pathophysiology itself, as studies have shown increased proliferation and migration of tumor cells after tumor biopsy, which included accelerated tumor growth in some cases [8,9]. Finally, the concept of determining therapy from a biopsy taken from a single location in the tumor may not accurately represent the tumor’s molecular characteristics, as many studies have shown immense heterogeneity throughout a GB, even on the single cell level [10,11].

There is a strong need for safely acquired and accurate information about the tumor molecular characteristics. Moreover, analysis of longitudinal samples would provide much needed insights in therapy responses and could provide opportunities to identify novel therapeutic targets. Today, disease progression and response to therapy can only be monitored by imaging techniques such as MRI, CT, and PET CT. Although these techniques can provide information about changes in a tumor mass, they too possess technique-specific drawbacks. For example, these imaging techniques are–to date–not able to reliably distinguish true tumor progression from pseudo-progression. Pseudo-progression is a phenomenon observed on MRI in 10–20% of patients during treatment of glioblastoma with Temozolomide and radiotherapy [12]. Therapy induced necrosis of glioma and endothelial cells causes edema and abnormal vessel permeability, mimicking tumor progression, which is indistinguishable from actual residual tumor growth. There is a great need for a reliable way to distinguish these two processes, as the different underlying conditions warrant different treatments. In addition, imaging techniques do not allow for assessment of changes in the molecular profile of a GB that have been shown to change in response to therapy or in the case of tumor recurrence [13,14]. The relative inaccessibility of the tumor hinders accurate follow-up of the molecular changes in GB during and after therapy, limiting our ability to adequately adapt our therapy.

One way to fulfill the need for longitudinal tumor sampling while minimizing risk of the patient is via liquid biopsies [15]. Analysis of tumor-derived EVs found in bodily fluids such as blood or cerebrospinal fluid (CSF), is a promising, minimally invasive, liquid biopsy strategy [16,17]. EVs are lipid bilayer enclosed structures of ~50–200 nm that bud from the cell membrane or are secreted from multi-vesicular endosomes [18,19]. The molecular cargo of EVs includes proteins, lipids, and RNA mimicking the phenotype of the releasing cell [20,21]. Cancer cells seem to be typically adept at releasing EVs and so-called oncosomes, large EVs shed from cancer cell membrane [22]. Analysis of circulating tumor EVs may therefore enable GB subtyping at the level of the whole tumor rather than the biopsied area alone [23]. Various efforts are being undertaken to use EVs isolated from blood or cerebrospinal fluid (CSF) as a biomarker in GB [24]. Although promising, one technical challenge is to identify and characterize the low number of tumor-derived EVs present in body fluids. Blood of a healthy volunteer contains approximately 10^10^ circulating EVs per ml plasma and these EVs are derived from many different cell types [25]. It has been shown that GB-derived EVs are present in the circulation, but the number of GB-derived EVs in the blood is probably low [26,27]. Strategies to detect and isolate GB-derived EVs are therefore required to further analyze the molecular contents of these tumor specific EVs.

Here we present an approach to detect, sort and characterize GB-derived EVs by using 5-aminolevulinic acid (5-ALA) induced fluorescence. 5-ALA (Gliolan), is an United States Food and Drug Administration (FDA) approved drug that is used to distinguish GB tissue from normal tissue during surgery. 5-ALA, a precursor to heme, is orally administered to the patient before surgery and taken up by glioma cells, where it is metabolized to fluorescent protoporphyrin IX (PpIX) in the mitochondria [28]. PpIX then accumulates in glioma cells due to decreased levels of ferrochelatase and selective uptake by ATP-binding cassette transporter ABCB6 [29]. Upon excitation with 405 nm wavelength light, the elevated levels of PpIX in glioma cells causes them to fluoresce in bright violet-red, enabling easier identification of malignant tissue during surgery to improve the chance of maximal surgical resection [30].

We show that administration of 5-ALA induces accumulation of PpIX in GB-derived EVs. This increased fluorescence in GB-derived EVs is detectable by high-resolution flow cytometry and allows for the detection of tumor EVs in the plasma of GB patients. Using advanced high-resolution flow cytometric sorting, we can isolate PpIX-positive EVs from the plasma of GB patients. With digital droplet PCR, we can detect tumor specific micro-RNAs in as few as 5 sorted PpIX-positive EVs. Our data illustrates the possibilities and limitations for fluorescent EV-based liquid biopsies in GB patients.

## 2. Materials and Methods

### 2.1. Platelet-Free Plasma Sample Processing

This study was approved by the ethics review committee of UMC Utrecht (protocols 18-020 and 18-021). Blood samples from 30 patients suspected of GB were collected. Per hospital protocol, 5-ALA was given orally 3 h before the start of surgery at a dose of 20 mg/kg. Before start of the surgery, arterial whole blood was taken in citrate buffered collection tubes and kept in the dark to avoid fluorescent quenching. The blood was then centrifuged at 1500× *g* for 10 min at room temperature. After the centrifugation, the top fraction of plasma was pipetted in standard 1.5 mL Eppendorf tubes and centrifuged at 13,000× *g* for 10 min at room temperature. The supernatants were then pooled, homogenized, and aliquoted in 1.5 mL Eppendorf tubes and stored at −80 °C for later use.

### 2.2. Cell Culture and Microscopy

The established cell line U87-MG/EGFRvIII (MG: Malignant Glioma, human GB cell line) was cultured in Dulbecco’s modified Eagle’s medium (DMEM) (Invitrogen, Carlsbad, CA, USA) containing 10% fetal bovine serum (FBS; Invitrogen) supplemented with penicillin (100 units mL^−1^) and streptomycin (100 μg mL^−1^; Invitrogen). The authenticity of the cell line was confirmed by short tandem repeat (STR) analysis, using the AmpFLSTR Identifiler PCR Amplification Kit (Applied Biosystems, Foster City, CA, USA). 24 h before microscopic experiments or EV isolation the medium was changed to medium without FBS and a 500 μM 5-ALA (Sigma-Aldrich, Darmstadt, Germany) concentration consisting of DMEM without phenol-red (Lonza, Basel, Switzerland) supplemented with penicillin (100 units mL^−1^) and streptomycin (100 μg mL^−1^; Invitrogen) and ultraglutamine (Lonza). All cell cultures were maintained at 37 °C in a humidified atmosphere of 5% CO_2_. For microscopic analysis 1.76 × 10^4^ cells were plated in chambered covered glass slides (Lab-Tek II) and placed on Zeiss Axiovert 200 M microscope using a set-up with 490–510 nm excitation and a dichroic BP585 nm emission filter (590–650 nm).

### 2.3. EV Isolation

A total of 1.54 × 10^6^ cells were plated in 15 cm cell culture plates for 24 h in complete FBS containing medium, after which the medium was switched to the 5-ALA containing medium described above and the cells were cultured for another 24 h. The supernatant was then collected and centrifuged at 300× *g* for 10 min to remove cellular debris. The subsequent supernatant was centrifuged at 2000× *g* for 10 min and then loaded in ultracentrifuge tubes and centrifuged at 100,000× *g* for 70 min (SW32 rotor, Beckman Coulter, Pasadena, CA, USA) after which the pellet was resuspended in phosphate buffered saline (PBS) and loaded in smaller ultracentrifuge tubes (SW41 rotor, Beckman Coulter, Brea, CA, USA) and centrifuged for an additional 70 min at 100,000× *g*. The EV containing pellet was then resuspended in PBS, aliquoted and stored at stored at −80 °C for later use. As PpIX signal is known to be susceptible to photobleaching [31], cells and samples were processed in the dark or under low light conditions as much as possible.

### 2.4. Tunable Resistive Pulse Sensing

EVs were quantified using tunable resistive pulse sensing as described before [32,33]. In brief, isolated EVs were diluted 1:10 in PBS and then loaded onto the qNano instrument (Izon Science Ltd., Christchurch, New Zealand). The samples were measured in duplicate alternating with 203 nm polystyrene calibration beads using an NP100 crucifix. Data was recorded and analyzed using the Izon Control Suite Software. The default minimum blockade height (0.05 nA) for particle detection was used.

### 2.5. High-Resolution Flow Cytometry

**Figure 1:**
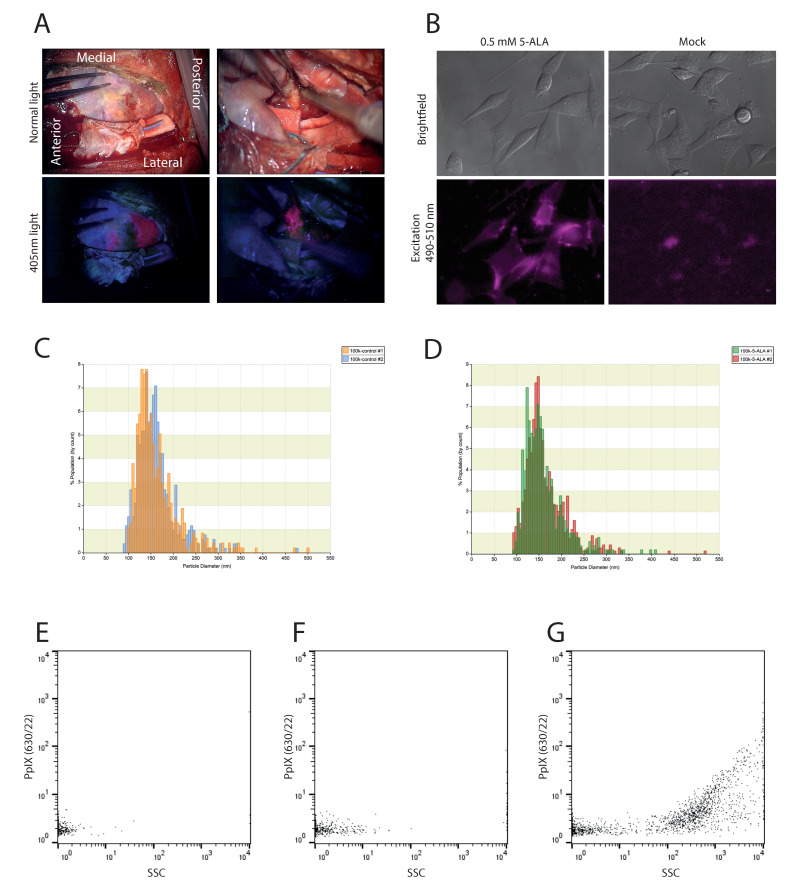
Administration of 5-ALA leads to PpIX fluorescence in vitro and in vivo. (**A**) Intra-operative view of Glioblastoma resection under white light (top row) and under 405nm light (bottom row). Protoporphyrin IX (PpIX) fluorescence is seen as pink. (**B**) U87 cells treated with 5-aminovulenic acid (5-ALA) (left panels) show PpIX fluorescence upon excitation at 490nm compared to control (right panels). 40× magnification. (**C**) Tunable resistive pulse sensing (TRPS) analysis of EVs isolated from U87 cells treated with 5-ALA. *n* = 2 (**D**) TRPS analysis of EVs isolated from U87 cells treated with PBS control. *n* = 2. (**E**) High-resolution flow cytometry (hFC) analysis of PBS. (**F**) hFC analysis of PBS with EVs isolated from U87 cells. (**G**) hFC analysis of PBS with EVs isolated from U87 cells treated with 5-ALA.

**Figure 2:**
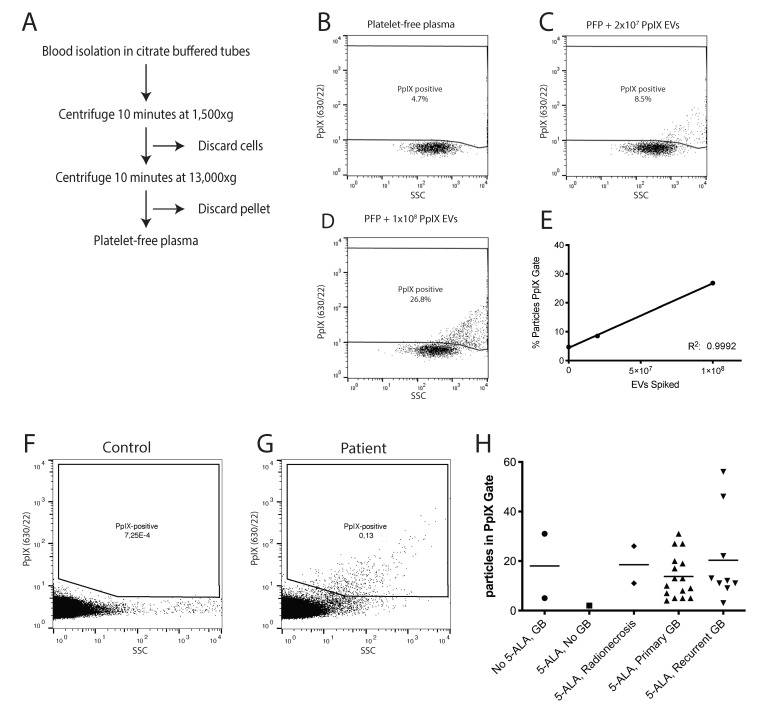
Isolation of PpIX-positive EVs from patient plasma. (**A**) Centrifugation protocol for the isolation of platelet-free plasma from whole blood. (**B**) hFC analysis of platelet-free plasma. (**C**) hFC analysis of platelet-free plasma spiked with 2 × 10^7^ U87 derived PpIX-positive EVs. (**D**) hFC analysis of platelet-free plasma spiked with 1 × 10^8^ U87 derived PpIX-positive EVs. (**E**) Linear regression plot of the % of particles in the PpIX gate versus the number of PpIX EVs spiked in. Goodness of fit, R^2^: 0.9998. (**F**) hFC analysis of PpIX particles in platelet-free plasma from control (10 min measurement). (**G**) hFC analysis of PpIX particles in platelet-free plasma from a patient treated with 5-ALA (10 min measurement). (**H**) Number of particles in the PpIX gate per patient group detected during 5 min of measurement.

**Figure 3:**
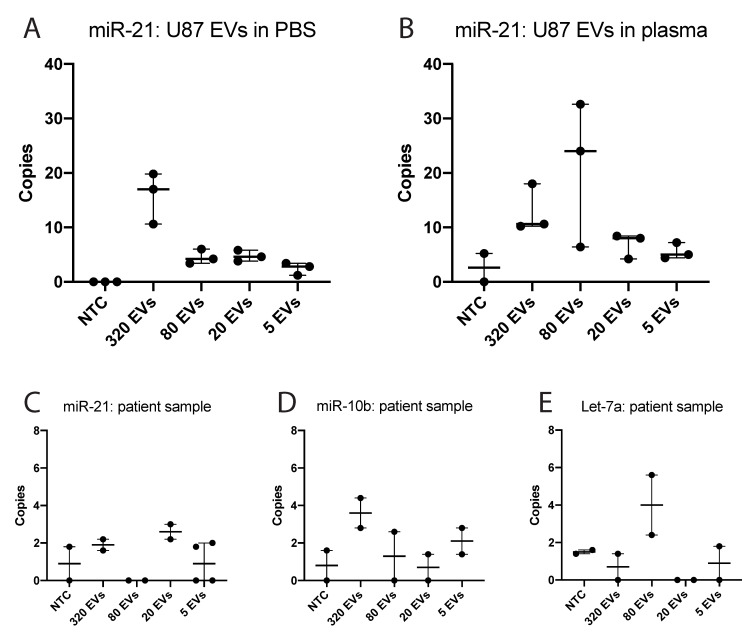
ddPCR analysis of PpIX-positive EVs. (**A**) Number of copies of miR-21 found in the indicated number of sorted PpIX-positive EVs from U87 cells treated with 5-ALA. EVs isolated by ultracentrifugation were diluted in PBS and sorted with high-resolution flow cytometry. NTC: non-template control. EVs: extracellular vesicles. (**B**) Number of copies of miR-21 found in the indicated number of sorted PpIX-positive EVs from U87 cells treated with 5-ALA. EVs were isolated by ultracentrifugation, diluted in healthy donor plasma, and sorted with high-resolution flow cytometry. (**C**) Number of copies of miR-21 in PpIX-positive EVs sorted from a patient with GB after receiving 5-ALA. (**D**) Number of copies of miR-10b in PpIX-positive EVs sorted from a patient with GB after receiving 5-ALA. (**E**) Number of copies of Let-7a in PpIX-positive EVs sorted from a patient with GB after receiving 5-ALA.

High-resolution flow cytometry of EVs was performed on a jet-in-air-based Becton Dickinson Influx flow cytometer (BD Biosciences, San Jose, CA, USA) using an optimized configuration as previously described in detail [34,35]. For experiments presented in this study, only the 405 nm laser was used. The optical configuration was modified to allow the collection of forward scattered light (FSC), side scattered light (SSC), and fluorescence off the 405 nm laser. To that end, a 405/20 bandpass filter was placed in front of the FSC detector combined with a 5 mm obscuration bar in front of the FSC collection lens to reduce excessive optical background [35]. Both PpIX fluoresce and SSC were collected at a 90 degree angle and in this direction a 480 long pass dichroic mirror was placed to reflect the shorter wavelength to the SSC detector equipped with a 405/20 bandpass and a neutral density (ND) filter. The PpIX fluorescence passed the dichroic mirror to reach a photomultiplier tube (PMT)equipped with a 630/22 bandpass filter. All scatter and fluorescence parameters were set to log scale. The PpIX fluorescence was used as a trigger signal and a threshold was set to reduce background signal (background set at less than 50 events per second) using clean PBS samples for cell culture EV (Figure 1), or healthy platelet-free plasma diluted 1:300 in PBS for detection of PpIX-positive EV in plasma (Figure 2 and Figure 3). The alignment of the Influx was performed using 8 peak Rainbow Calibration Particles (Biosciences, Erembodegem, Belgium). For high-resolution analysis of the samples, a nozzle with a bore size of 140 µM was used. Pressure of sheath (4.89–5.02 PSI) and sample (4.29 PSI) were kept constant to allow identical diameter of sample core in the jet stream during for the entire measurement.

All samples were prepared in PBS. Platelet-free plasma samples were diluted 1:300 prior to analysis. All samples were measured in a fixed time window (30 s for EVs from in vitro cultured cells and 300 s for plasma sample) to allow for comparison of EV numbers between samples. To calculate the number of PpIX-positive events in plasma samples, a gate was placed based on SSC and PpIX fluorescence values or SSC-PpIX fluorescence allowing a maximum of 10 events in this gate for healthy plasma run as a control. Measurements were performed using BD FACS Software 1.01.654 (BD Biosciences).

### 2.6. High-Resolution Flow Cytometric Sorting of EVs

For sorting experiments, the system and procedure were optimized to allow for maximal event rates and small sort volumes, but to avoid coincidence [36]. In short, a 70 μm nozzle was used and sheath fluid pressure increased to 30 psi. At a sample fluid pressure of 29 psi, an event rate of ≤5000 per second was reached (higher event rates resulted in coincidence and swarm). The drop frequency was 49.22 kHz with sort efficiencies of approximately 95%. Drop delay was adjusted using Accudrop beads (Cat nr.: 345249 BD Biosciences). Drop delay time was cross checked by sorting these beads on microscope glass slides using the coarse calibration slide sorting mode of the BD Influx and if necessary adjusted to the proper delay time. For EV sorting, plasma samples were diluted 1:300 in PBS. Sorted EVs were collected in 96 well plates at 5 to 320 EV per well. The volume of one sorted drop was calculated to be 2 nl.

### 2.7. Measurement of miRNA Levels from Sorted EVs

Reverse transcription was performed using the TaqMan miRNA Reverse-Transcription Kit and miRNA-specific stem-loop primers (Applied BioSystems) in a 15 μL RT reaction (comprised of 8.56 μL of H_2_O, 1.5 μL of 10× Reverse-Transcription Buffer, 0.19 μL of RNase-Inhibitor (20 units/L)), 1 μL of 100 mM dNTPs with dTTP, 1 μL of Multiscribe Reverse Transcriptase, and 3 μL RT primer [miR16 (RT:000391); miR10b (RT:002218); let7a (RT:000377); miR21 (RT:000397); Catalog: 4427975 Thermofisher]. These components are prepared as a larger master mix and 14.4 μL of master mix is added to each well of a 96 well plate with the sorted EVs and mixed well by pipetting. RT reactions are carried out in a Tetrad2 Peltier Thermal Cycler (Bio-Rad, Hercules, CA, USA) at 16 °C for 30 min, 42 °C for 30 min and 85 °C for 5 min and held at 4 °C. For droplet digital PCR analysis, 5 μL of cDNA was added to the reaction mixture containing 10 μL of 1× ddPCR Supermix for probes (no dUTP, Bio-Rad), 1 μL of probes 20× primer [miR16 (TM:000391); miR10b (TM:002218); let7a (TM:000377); miR21 (TM:000397); Catalog: 4427975 Thermofisher] and 4 μL of water to yield a total reaction volume of 20 μL. The QX200 AutoDG Automatic Droplet generator (Bio-Rad) was used to generate droplets. Thermocycling conditions were as follows: 95 °C (51% ramp) for 10 min, 40 cycles of 94 °C (51% ramp) for 30 s and 60 °C for 1 min, followed by 98 °C for 10 min and held at 4 °C until further processing. Droplets were counted and analyzed using the QX200 droplet reader (Bio-Rad) and QuantaSoft analysis (Bio-Rad) was performed to acquire data and later normalized to the input.

### 2.8. Statistical Analysis

Graphing and statistical analysis was done in Prism version 8.3.0 (Graphpad Software LLC, San Diego, CA, USA). Figures were prepared in Adobe Illustrator 2019 (Adobe Systems, San Jose, CA, USA).

## 3. Results

### 3.1. Administration of 5-ALA Leads to Accumulation of Fluorescent PpIX in EVs

Prior to neurosurgery, GB patients are administered 20 mg/kg 5-ALA orally. In most patients, tumor cells can be detected during surgery by their fluorescent pinkish glow upon excitation with a 405 nm laser (Figure 1A). To replicate this effect in vitro, we cultured human glioma U87-MG/EGFRvIII cells in vitro with or without 5-ALA. We selected 500 μM as an optimal 5-ALA concentration at which cells were efficiently labeled and remained viable (data not shown). This is in line with previous publications on 5-ALA labeling of in vitro cultured cells [28,31]. As expected, accumulation of fluorescent PpIX could be detected in cells treated with 5-ALA for 24 h (Figure 1B). To evaluate whether PpIX also accumulates in EVs, we isolated EVs from the supernatant of the cultured cells by differential centrifugation. Using tunable resistive pulse sensing (TRPS) we could detect particles in the 100–200 nm size range, as expected for EVs [32,37]. Similar size distribution and EV concentrations were observed from both the 5-ALA treated cells as for the mock control cells (Figure 1C,D). To determine if EVs harbor fluorescent PpIX, both the mock control and 5-ALA treated EVs were analyzed by high-resolution flow cytometry (hFC) [34,35] by thresholding on the PpIX fluorescence signal. Our data indicated that EVs from 5-ALA treated cells contained sufficient fluorescence to be detected above the fluorescence threshold, while EVs from the control cells were not detectable (Figure 1E–G). Together, these results indicate that treatment of in vitro cultured glioma cells with 5-ALA can induce the loading of fluorescent PpIX in glioma cell-derived EVs. Individual PpIX-positive EVs can be detected using high-resolution flow cytometry.

### 3.2. PpIX-Positive EVs Can Be Detected in the Plasma of Glioblastoma Patients

Until now, hFC has only been applied to analyze EVs that were labeled after purification from body fluids. Our next goal was to evaluate whether hFC could be used to detect individual PpIX EVs against the background of unfractionated patient plasma. Plasma is a protein-rich fluid that contains many macromolecular structures such as lipoparticles. All these structures likely contribute to the background signals during hFC. To optimize hFC settings, we used platelet-free plasma isolated from whole blood from healthy donors via centrifugation (Figure 2A). Fluorescence threshold triggering was applied on the channel used to detect PpIX fluorescence. We first performed serial dilution of the plasma to find the dilution at which hFC analysis was not affected by (invisible) swarm [38]. We raised the threshold until less than 50 background events per second were visible. Next, we analyzed plasma samples in which we spiked different numbers of U87-5ALA EVs. Single PpIX-positive particles could be detected above the background signal in a dose dependent manner (Figure 2B–E).

To determine if PpIX-positive particles could be detected in the platelet-free plasma of GB patients, we collected samples from patients who underwent surgery for suspected GB using 5-ALA as an intra-operative tool. In this group, two GB patients undergoing surgery without the use of 5-ALA were also included. Two other patients underwent 5-ALA assisted surgery, but histopathological analysis of the suspected mass did not show the presence of malignant cells (one patient was diagnosed with a chronic inflammation and one patient with radiation induced changes). These two groups were used as controls. All other included patients had either primary or recurrent WHO IV glioblastoma, as determined by post-operative histopathological analysis. All patients with recurrent GB had previously been treated according to the Stupp protocol [39] (resection followed by 6 weeks of radiotherapy (total of 60 Gy)) combined with Temozolomide (75 mg per square meter body-surface area), followed by six cycles of adjuvant Temozolomide (150 to 200 mg of Temozolomide per square meter for 5 days during each 28 day cycle). Additional patient characteristics can be found in Table S1. Blood was collected after 5-ALA administration, just before start of the surgery. The number of PpIX-positive EVs was low with 5–60 EVs per patient collected during an acquisition time of 300 s in which a total of 1 ul of plasma diluted in PBS was analyzed. The number of positive events varied widely between samples, with one control having a considerate number of particles in the PpIX gate (Figure 2H). We had limited data on radionecrosis, but this also appeared to enhance PpIX fluorescence. The number of PpIX-positive particles was highest in patients with recurrent GB. Figure 2F,G illustrate the detection of PpIX-positive EVs in a healthy control and patient sample, ran for a longer time period than the patients in Figure 2H for illustrative purposes. These data suggest that PpIX-positive EVs can be detected in patient plasma with GB but that the levels are highly variable.

### 3.3. PpIX-Positive EVs Isolated by High-Resolution Flow Cytometric Sorting Contain GB-Associated miRNAs

We previously showed that our hFC system allows sorting of single EVs harboring fluorescence or light scattering characteristics of interest [36,40]. The number of PpIX-positive, GB-derived EVs in patient plasma is low (Figure 2H). Using high-resolution flow cytometric sorting (see Figure S1B,C for the gating strategy), we were able to isolate PpIX-positive vesicles, To further evaluate these particles, we performed ddPCR for several GB-associated and EV associated miRNAs on the isolated particles.

To test the feasibility of detecting tumor-related miRNAs in the low numbers of sorted EVs, we first sorted increasing numbers of PpIX-positive EVs (5–320 EV) from 5-ALA treated U87-MG/EGFRvIII cells in a 96 well plate and performed ddPCR for miR-21 (Figure 3A). We were able to detect copies of miR-21 in as little as five EVs. We then spiked PpIX-positive EVs from 5-ALA treated U87-MG/EGFRvIII cells into plasma from a healthy donor and sorted increasing numbers of EVs for ddPCR analysis. The number of detected miR-21 copies in these EV isolates was more variable, but signal was detectable above negative control levels *(*Figure 3B). Sorting higher numbers of EV per sample led to a decrease, rather than an increase, in the number of detected miRNA copies (Figure S1D,E). We speculate that the increased volume of buffer in which higher numbers of EVs are sorted interferes with the RNA isolation efficiency and/or ddPCR.

After optimizing our set-up we quantified the presence of three miRNAs in PpIX-positive EVs sorted from a GB patient treated with 5-ALA. miR21, an antiapoptotic factor, is highly expressed in GB [41] and GB EVs [42] as is miR10b, which is associated with tumor invasive properties and glioma growth [43,44]. Let7a is aberrantly expressed in GB and can be used as a marker for EVs [45,46]. We confirmed that multiple copies of these miRNAs could be detected in the PpIX-positive EVs of a GB patient treated with 5-ALA (Figure 3A). Although the levels of the miRNAs detected in the EVs were low and variable, the signal was above the control (no template control, NTC). No clear relationship was observed between the number of EVs sorted and the miRNA copy number. The heterogeneity of EVs and their cargo could explain this observation [20], but certain technical limitations may also play a role. This data shows that hFC sorting of EVs and subsequent analysis with ddPCR is feasible, illustrating the potential of this method in identifying circulating GB-derived EVs based on PpIX fluorescence.

## 4. Discussion

For several years, 5-ALA has been used in GB patients for intra-operative tumor tissue visualization [28,47]. Here we show that after administration of 5-ALA, PpIX accumulates in glioma derived EVs both in the media of cell cultures and in the plasma of GB patients. Using high-resolution flow cytometry, we were able to detect and isolate PpIX-positive, GB-derived EVs. Sorting these EVs allowed us to analyze their contents, which confirmed the presence of GB specific miRNA miR-21 [41], miR-10b [43] and EV specific miRNA (let-7a) [46].

Given the challenges associated with (repeated) surgery for tissue diagnosis, establishing a reliable liquid biopsy technique is a major goal in glioma research. Various approaches are being explored, including isolation of circulating tumor cells (CTCs), circulating tumor DNA, and EVs [15]. EVs may be more suitable as biomarkers for glioblastoma as they are more abundant than CTCs [48] and because the genetic content of EVs is relatively more stable compared to circulating DNA due to enclosure in lipid bilayers [49]. The major challenge is to isolate GB-derived EVs from the billions of circulating particles in the blood [25]. Most attempts to isolate GB-derived EVs from the circulation include selection of EVs by capturing specific surface proteins [50]. So far none of these approaches have made it into clinical practice and recent evidence that the ectodomain of EV-surface receptors can be cleaved off may further complicate this approach [51].

We investigated an approach of using a byproduct of a clinically approved drug currently used during glioma surgery to detect EVs in the circulation of GB patients. The idea that oral administration of 5-ALA might lead to not only PpIX accumulation in tumor cells but also in tumor-derived EVs has been proposed previously [52]. Recently, Jones et al. showed that PpIX-positive EVs are released by glioma cells treated with 5-ALA in vitro, and can be detected in the plasma of glioma xenograft mice and patients who had been administered 5-ALA prior to surgery, using imaging flow cytometry [53]. Here, we show for the first time the ability to not only detect these PpIX-positive EVs, but also to isolate them using high-resolution flow cytometric sorting for downstream analysis. This is a major step in the development towards liquid biopsies in GB to isolate the tumor specific EVs and to decrease the necessity of high throughput modalities such as next-generation sequencing for analysis.

Before this technique can be applied at a larger scale, several technical hurdles need to be overcome. The signal-to-noise ratio for hFC-based detection of PpIX-positive particles against background signals from plasma is low. This may explain why even in patients that did not receive 5-ALA, some positive particles are detected. Reduction of plasma protein background could be achieved by including a size exclusion chromatography step, but potential loss of PpIX-positive EVs in the column would impose a substantial risk to reduce accuracy in the rare event analysis. Our high-resolution flow cytometry approach did not provide information on the total number of EV in the sample, as particles were only registered if their PpIX fluorescence exceeded the threshold set by the user. The percentage of PpIX EVs respective to the total number of EVs could therefore not be measured, but instead an absolute number of positive events in a volume or run time was obtained. It should also be noted that the gating and sorting of the PpIX-positive EVs is tedious and strict protocols should be followed to prevent swarm effects, which can interfere with the results [36].

Large variability was observed in the number of detectable PpIX-positive EVs between patients and controls. PpIX-positive particles were observed in two patients who did not receive 5-ALA prior to surgery, but who did have active GB. As we know that PpIX accumulation occurs in GB and 5-ALA exacerbates this effect, it could be that even without 5-ALA some particles fluoresce with PpIX. As our technique is extremely sensitive, it is possible that we pick up these few particles. Another explanation can be found in several (rare) syndromes that have been linked to PpIX accumulation without 5-ALA administration, such as X-linked protoporphyria and human erythropoietic protoporphyria [54]. More common mechanisms such as iron deficiency and the use of nonsteroidal anti-inflammatory drugs have also been linked to PpIX accumulation in cells [55]. In patients who did receive 5-ALA, PpIX fluorescence can also occur in non-tumor tissue [56,57]. One study showed that in 13 out of 313 cases operated on for recurrent GB, the PpIX fluorescent material that was resected showed only reactive tissue with notable edema, inflammatory cell infiltration and presence of reactive astrocytes [57]. In the two patients who turned out to have radio necrosis in our cohort, we also picked up PpIX-positive particles, supporting this data. Radionecrosis, disruption of the blood-brain barrier and infiltration of inflammatory seemed to play a role in this process, but the exact relation with accumulation of PpIX in EVs remains to be understood. Given the various possible sources of PpIX-positive EVs, it is possible that some of the particles we observed in patients with GB are derived from non-GB tissues. However, the presence of the GB enriched miR-21 and miR-10b suggests that these particles have GB origin. EVs are known to contain very little miRNAs, averaging less than one non-ribosomal RNA per EV [58]. The presence of miR-21 and miR-10b, both highly expressed in GB but not in normal tissue [41,42,43], could be an indicator for GB origin. To provide a more complete overview and comparison, we aim to further analyze PpIX-positive EVs from GB patients and non-GB patients in future studies with various newer sequencing techniques.

Our in-house developed method to perform hFC-mediated sorting of single EVs in ultra-low volumes (2 nl drop volume) allowed for ddPCR analysis on exact numbers of collected EVs. The copy number of miR-21 in EVs isolated from U87 cells roughly resembled the number of EVs sorted, while in patient data the number of miRNA copies was much more variable (Figure 3). Although it is possible that the quantitative sorting process was not 100% accurate, another likely explanation is that the population of PpIX-positive EVs is heterogeneous in volume and molecular cargo. EVs derived from U87 cells in vitro are likely more homogeneous in content compared to those from patient gliomas. Heterogeneity is a common feature of EV populations derived from glioma patients [20,59,60], as is heterogeneity of glioma tissue itself [10]. Furthermore, the EVs measured in vitro varied in diameter from 100 to 200 nm. For spherical EVs, this implies a difference in volume of factor 8 between the largest and smallest EVs detected. As a result, the small groups of individually sorted EVs may contain variable copy numbers of the tested miRNAs and, in the limited number of EVs sorted, a linear relation between EVs and copy numbers is not to be expected. Our efforts to analyze a larger number of EVs with ddPCR were limited due to a loss of signal with increased input (Figure S1D,E). It is vital that these techniques for downstream analysis are optimized in future studies. Additionally, improved ddPCR analysis could serve as a secondary method to detect ‘false positives’, EVs that were PpIX-positive but did not have a GB origin.

The number of PpIX fluorescent particles in GB patients differed widely. Part of this variation may be explained by differences in tumor fluorescence, which depends on many variables, including time between 5-ALA administration and surgery, necrosis [28], glioma subtype [61] and proliferation index [62]. To what extent these factors contribute to the number of PpIX-positive events remains to be elucidated, as our study was underpowered to evaluate these variables. Furthermore, PpIX signal bleaches quickly under white light [31,53], so precautions have to be taken to prevent loss of signal. We evaluated the change in signal in respect to time from sample collection to EV isolation, but found no negative correlation. More reliable results may also be obtained by comparing the number of PpIX-positive particles in plasma samples from patients collected during surgery to the number observed in pre-surgery samples, which can be used as in-patient controls [53]. In future studies, more controls and patient data should be obtained to evaluate whether other factors could cause EVs to carry a PpIX(-like) signal. Given the sensitivity of PpIX fluorescence to bleaching, we applied stringent standardization of blood acquisition with rapid plasma processing, as these steps may influence the number of background events. We strongly encourage researchers to take these precautions when working with PpIX fluorescence in GB.

The data presented here illustrate the potential of identifying and sorting PpIX EVs to uncover differences in expression profile between the various GB cells using GB patient plasma as a liquid biopsy.

## 5. Conclusions

Our data shows the potential for isolating GB-derived PpIX-positive EVs from plasma after oral administration of 5-ALA via high-resolution flow cytometric sorting. Although several challenges remain, we have taken yet another step on the road towards improving GB diagnostics using EVs in liquid biopsies. With further development and refinement, these techniques will open various avenues of longitudinal follow-up, disease monitoring, customization of therapy and, possibly, screening for either recurrence or primary disease in patients at risk. By evading invasive surgical biopsies, this technique enables researchers to gain new insights in GB pathogenesis and will aid in improvements of care and treatment for patients suffering from glioblastoma.

## Supplementary Materials

The following are available online at https://www.mdpi.com/2072-6694/12/11/3297/s1, Table S1: Patient characteristics, Figure S1: (A) Example of the gating strategy employed for sorting of PpIX-positive EVs in a healthy control and (B) in a GB patient treated with 5-ALA. (C) ddPCR detailing the number of copies of miR-21 found in various numbers of U87 EVs sorted from PBS. (D) EVs derived from U87 cells were spiked in healthy plasma and consequently sorted with high-resolution flow cytometry. ddPCR for miR-21 was performed on various numbers of EVs.
