# Supplementary material for: Orally Administered 5-aminolevulinic Acid for Isolation and Characterization of Circulating Tumor-Derived Extracellular Vesicles in Glioblastoma Patients
Source: Cancers (Basel). 2020 Nov 7;12(11):3297. doi: 10.3390/cancers12113297 (PMC7695169; doi:10.3390/cancers12113297)
Supplement: Supplementary file 1 [file cancers-12-03297-s001.zip › Supplementary Table 1.docx]

| **Supplementary Table 1a: Patient characteristics** | | | | | | | |
| --- | --- | --- | --- | --- | --- | --- | --- |
| **Study ID** | **Age** | **Sex** | **Post-operative**  **diagnosis** | **Location of tumor** | **Estimated tumor size (cm3)** | **5-ALA administered** | **PpIX positive events** |
| 1 | 66 | female | GB | right-sided frontal lobe | NA | No | 5 |
| 2 | 67 | female | GB | right-sided temporal lobe | NA | No | 31 |
| 3 | 65 | male | GB | left-sided central | 14 | Yes | 9 |
| 4 | 73 | male | GB | left-sided temporal lobe | 113 | Yes | 13 |
| 5 | 65 | female | GB | butterfly, frontal lobes | NA | Yes | 31 |
| 6 | 65 | female | GB | left-sided frontal lobe | 8 | Yes | 5 |
| 7 | 68 | male | GB | right-sided parietal-temporal lobe | NA | Yes | 5 |
| 8 | 69 | female | GB | right-sided frontal lobe | NA | Yes | 27 |
| 9 | 77 | male | GB | left-sided temporal lobe | 34 | Yes | 17 |
| 10 | 66 | male | GB | right-sided frontal lobe | 34 | Yes | 4 |
| 11 | 66 | male | GB | left-sided parietal lobe | 14 | Yes | 8 |
| 12 | 66 | male | GB | left-sided frontal lobe | 34 | Yes | 7 |
| 13 | 60 | male | GB | left-sided frontal lobe | 45 | Yes | 13 |
| 14 | 60 | female | GB | left-sided temporal lobe | NA | Yes | 5 |
| 15 | 67 | male | GB | right-sided temporal lobe | NA | Yes | 19 |
| 16 | 73 | female | GB | right-sided temporal lobe | NA | Yes | 27 |
| 17 | 69 | female | GB | left-sided fontal lobe | 18 | Yes | 20 |
| 18 | 62 | male | GB | NA | NA | Yes | 10 |
| 19 | 44 | female | Recurrent GB | right-sided frontal lobe | NA | Yes | 11 |
| 20 | 43 | female | Recurrent GB | left-sided fontal lobe | NA | Yes | 13 |
| 21 | 72 | male | Recurrent GB | left-sided temporal lobe | NA | Yes | 46 |
| 22 | 41 | male | Recurrent GB | right-sided frontal lobe | NA | Yes | 9 |
| 23 | 52 | male | Recurrent GB | right-sided frontal lobe | 16 | Yes | 3 |
| 24 | 53 | male | Recurrent GB | NA | NA | Yes | 22 |
| 25 | 65 | male | Recurrent GB | right-sided frontal lobe | NA | Yes | 12 |
| 26 | 70 | male | Recurrent GB | right-sided temporal lobe | NA | Yes | 56 |
| 27 | 64 | male | Recurrent GB | left-sided occipital lobe | 7 | Yes | 11 |
| 28 | 62 | male | Radiation necrosis | left-sided temporal lobe | NA | Yes | 26 |
| 29 | 69 | female | Radiation necrosis | left-sided frontal lobe | NA | Yes | 11 |
| 30 | 59 | male | Chronic infection | right-sided occipital lobe | NA | Yes | 2 |

| **Supplementary Table 1b: Patient characteristics continued** | | | | |
| --- | --- | --- | --- | --- |
| **Study ID** | **Dexamethason dose prior to surgery (mg/day)** | **Oncological history** | **Pre-existing conditions at time of surgery** | **Medication at time of surgery** |
| 1 | 4 | none | Asthma, hypothyroidism | acetaminophen, levetiracetam, midazolam |
| 2 | 0 | Melanoma. Treatment unknown | none | dexamethasone, pantoprazole, citalopram, temazepam, betametason |
| 3 | 6 | none | none | hydrocxocobalamin, metformin, pantoprazole, acetaminophen, dexamethasone |
| 4 | 6 | none | Arrythmias | dexamethasone, hydrochlorothiazide, simvastatin, budesonide, esomeprazole |
| 5 | 6 | none | none | thyrax, vaseline, rosuvastatin, pantoprazole, acetaminophen, dexamethasone |
| 6 | 6 | pituitary macroadenoma. Resection, radiation. | none | asasantin, perindopril, simvastatin, salbutamol |
| 7 | 0 | prostate cancer | TIAs, COPD | dexamethasone, irbesartan/hydrochlorothiazide |
| 8 | 12 | none | HT | dexamethasone |
| 9 | 8 | Meningioma, WHO Grade I. Resection | HT, hypercholesterolemia, stomach resection (non-malignant), | simvastatin, tolbutamide, metformin, valsartan, masoprazole, dexamethasone, levetiracetam |
| 10 | 4 | Colon cancer. Resection and radiation | HT, DM2, ileostoma | dexamethasone, pantozole, acetaminophen, tramadol |
| 11 | 8 | none | HT, cholelithiasis | dexamethasone, pantoprazole, tamsulosin |
| 12 | 12 | none | HT, benign prostatic hyperplasia | depakine |
| 13 | 0 | none | none | dexamethasone, pantroprazole, simvastatin |
| 14 | 4 | none | hypercholesterolemia | dexamethasone, finasteride, hydrochlorothiazide, atorvastatin, levetiracetam, metoclopramide, acetaminophen, midazolam |
| 15 | 4 | none | HT | metformin, metoprolol, glimeperide, lercadipine, dexamethasone, atorvastatin, temazepam |
| 16 | 8 | none | HT, DM2, hypercholesterolemia | depakine, dexamethasone, stesolid, temazepam, oxazepam |
| 17 | 6 | none | HT, hypercholesterolemia | NA |
| 18 | NA | NA | NA | zopiclon, dexamethasone, oxazepam, ibuprofen, acetaminophen, omeprazole, movicolon, calciumcarbonate/colecalciferol, alendronic acid |
| 19 | 4 | GB. Resection, TMZ and radiation | none | tobramycin ophthalmic solution, valproinic acid, dexamethasone |
| 20 | 8 | GB. Biopsy, TMZ and radiation | Stevens-Johnson syndrome | candesartan, levetiracetam, metoprolol, acetaminophen, dexamethasone |
| 21 | 6 | GB. Resection, TMZ and radiation | HT, arrythmias | dexamethasone, pantoprazole, levetiracetam, midazolam, lisinopril/hydrochlorothiazide, magnesiumhydroxide |
| 22 | 8 | Anaplasic astrocytoma. Resection, TMZ and radiation | HT, DM2 | mometasone |
| 23 | 0 | GB. Resection, TMZ. | none |  |
| 24 | NA | NA | NA | atorvastatin, dexamethasone, acetaminophen, novorapid, omeprazole, salbutamol, tiotropium |
| 25 | 8 | GB. Resection, TMZ and radiation | COPD, femoral iliac bypass, DM2 | amlodipine, omeprazole, levetiracetam, budesonide, midazolam |
| 26 | 12 | GB. Resection, TMZ and radiation | atrial fibrillation | acetaminophen, Augmentin, tramadol, ipratropium, sertraline, salmeterol/fluticasone, levetiracetam, tiotropium, prednisolone, omeprazole, fentanyl, atorvastatin, clopidogrel |
| 27 | 0 | GB. Resection, TMZ and radiation | TIAs, COPD, | hydrochlorothiazide |
| 28 | 0 | none | HT | diazepam, levetiracetam, vitamin b, glucomotion, treamterene/epitizide, codine, acetaminophen, salbutamol, ipratropium, fluticasone, magnesiumhydroxide, omeprazole, dexamethasone |
| 29 | none | none | HT, hypercholesterolemia, psoriasis, TIA | levetiracetam, temazepam, acetaminophen |
| 30 | NA | none | none | NA |
