# Supplementary figures and images for: Orally Administered 5-aminolevulinic Acid for Isolation and Characterization of Circulating Tumor-Derived Extracellular Vesicles in Glioblastoma Patients
Source: Cancers (Basel). 2020 Nov 7;12(11):3297. doi: 10.3390/cancers12113297 (PMC7695169; doi:10.3390/cancers12113297)

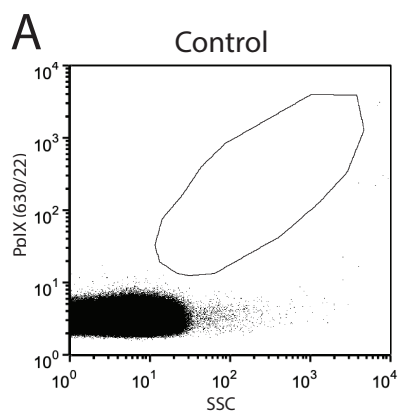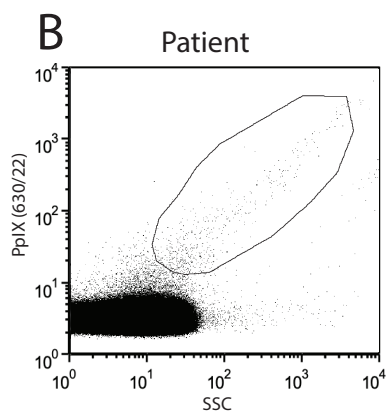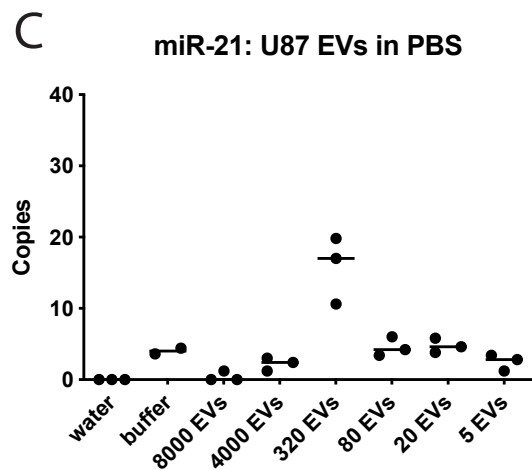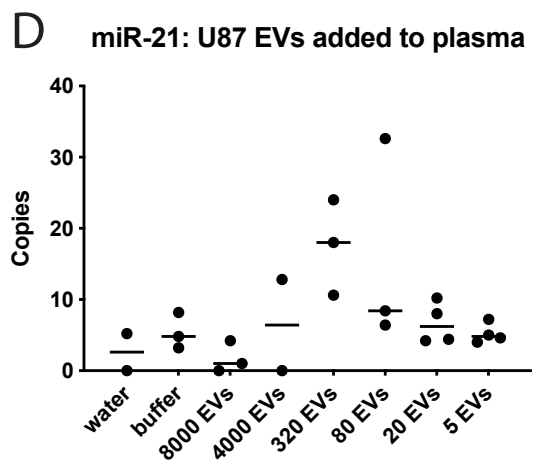

Supplement: Supplementary file 1 [file cancers-12-03297-s001.zip › SuppFig1.pdf]
